# Effects of Hydrogen Contents on Oxidation Behavior of Alloy 690TT and Associated Boron Accumulation within Oxides in High-Temperature Water

**DOI:** 10.1155/2018/7845176

**Published:** 2018-06-10

**Authors:** Soon-Hyeok Jeon, Geun Dong Song, Do Haeng Hur

**Affiliations:** Nuclear Materials Research Division, Korea Atomic Energy Research Institute, 989-111 Daedeok-daero, Yuseong-gu, Daejeon 305-35, Republic of Korea

## Abstract

The aim of this work is to characterize the oxide layer structure of Alloy 690TT in high-temperature water with different dissolved hydrogen (DH) contents by using an X-ray photoelectron spectroscopy. Under the low DH contents (0.4494–0.8988 mg/kg), the oxide layers were composed of an outermost layer of Ni(OH)_2_ and Cr(OH)_3_ enriched in Ni, an intermediate layer of hydroxides and oxides enriched in Cr, and an inner Cr_2_O_3_ layer. Outermost NiO coexists with small amount of Cr_2_O_3_ layer, while in the inner oxide only Cr_2_O_3_ remains. The oxide layers at medium and high DH contents (3.1458– 8.9880 mg/kg) consisted of an outermost layer of Ni(OH)_2_ and Cr(OH)_3_ enriched in Cr, an intermediate layer of metallic Ni, hydroxides and oxides enriched in Cr, and an inner Cr_2_O_3_ layer. In addition, boron compounds containing B^3+^ ions were accumulated in the thick and porous NiO layer formed at low DH contents, whereas the accumulation of boron compounds did not occur in the thin and dense polyhedral oxide layer formed at medium and high DH contents.

## 1. Introduction

Alloy 690, a nickel-based alloy having high chromium content (27–31 wt.%), was developed as a replacement for Alloy 600 steam generator (SG) tubes owing to its excellent resistance to stress corrosion cracking (SCC) in a pressurized water reactor (PWR) [1–4]. Although no in-service SCC failures of Alloy 690 tubes have been reported, its long-term service performance is a highly concerned issue because Alloy 690 still suffers from general corrosion in the primary water of PWRs [5]. The oxidation behavior of Alloy 690 has been one of the interesting topics because the oxide layers formed on alloys act as an important role to their corrosion resistance.

The effects of various water chemistry parameters such as dissolved oxygen (DO) [6], pH [7, 8], temperature [8–10], Li/B coordination [11], and Zn injection [12, 13] on the oxidation behavior of nickel-based alloys have been investigated extensively. Besides, dissolved hydrogen (DH) is also an important parameter because it can significantly affect the oxide formation and the corrosion behavior of nickel-based alloys [14–17].

There are many researches related to hydrogen contents in the primary water of PWRs. For the fuel cladding, Kawamura [18] showed that the amount of crud (Chalk River unidentified deposits) deposition and boron accumulation of the fuel cladding was not affected by a change in hydrogen content. Baek et al. [19] also presented that the DH content did not greatly affect the amount of crud deposits formed on the fuel cladding. In the viewpoint of nickel-based alloys, hydrogen-related researches have been focused mainly on the stress corrosion cracking (SCC). The DH content can significantly affect the crack growth rate (CGR) of nickel-based alloys in the primary water of PWRs [20, 21]. That is, the CGR shows a maximum value at a DH content where the Ni/NiO phase transition occurs. Therefore, it has been generally considered that the oxide layers formed on the surface of nickel-based alloys have a crucial role in the SCC growth rate [16]. Recently, it has been reported that the structure of oxide film of Alloy 690TT was significantly changed and general corrosion decreased with increasing the DH contents (0.4494–8.9880 mg/kg) [22]. In addition, it has shown that the oxide film formed on Alloy 690TT was porous and thick at low DH content (0.4494 mg/kg) in primary water system [22].

Meanwhile, boron accumulation may occur within not only the crud formed on the fuel cladding but also the oxide layer on Alloy 690TT at low DH content because both oxide layers have a thick and porous morphology. However, the effects of the hydrogen contents on the oxide structure and associated boron accumulation within oxide layer of Alloy 690TT have not yet been investigated. Furthermore, the detailed depth profile of the chemical state of the oxide layers formed on Alloy 690TT has not been reported under the broad range of DH contents by using X-ray photoelectron spectroscopy (XPS).

Therefore, the purpose of this study is to characterize the oxide layer structure of Alloy 690TT and associated boron accumulation within its oxide layers in primary water at 330°C and 150 bars in the broad range of DH contents (0.4494–8.9880 mg/kg) by using XPS. To observe the boron accumulation within the oxide layers, the depth profile and chemical species of the oxide layers were analyzed by XPS analysis.

## 2. Experimental

### 2.1. Specimen Preparation

Alloy 690TT tubes, which are commonly used as a SG tubing material, were used in this study. Alloy 690TT tubes have a dimension of a 19.07 mm outer diameter (OD) and a 16.93 mm inner diameter (ID). The specimens were cut into a size of 10 mm × 10 mm × 1.07 mm for the oxidation tests and various oxide layer analyses. The specimens were cleaned in acetone and distilled water. After the specimens had been cleaned, the piece specimens were directly dried in a vacuum oven at 60°C. While the preparing for the oxidation tests, the samples were stored in a vacuum desiccator at room temperature to prevent the oxidation reaction in the air. In addition, because Alloy 690TT is a high-alloyed steel having excellent corrosion resistance, the surface composition of the Alloy 690TT before the oxidation test could be considered to the same as that of the matrix. The chemical composition of Alloy 690TT was analyzed in accordance with the standard of ASTM E353-14 for method for chemical analysis of stainless, heat-resisting, maraging, and other similar chromium-nickel-iron alloys [23]. The chemical composition of Alloy 690TT tubes is given in Table 1.

### 2.2. Oxidation Tests

Oxidation tests for the oxide formation were performed in an autoclave connected to a high-temperature water circulating system. The circulation system consisted of the following main components: a primary solution tank, high-pressure pump, preheater for solution inlet temperature control, back pressure regulator for pressure control, heat exchanger, and an autoclave for placing the specimens. The test solution was preheated and entered into the autoclave where the piece specimens were hung on a specimen holder.

The test solution was maintained consistently at 330°C under 150 bars in the autoclave because the saturation pressure of the high-temperature water at 330°C is about 128 bars. The simulated primary water was made using the demineralized water with the resistivity above 18 M*Ω*·cm, nuclear-grade lithium hydroxide (LiOH), and boric acid (H_3_BO_3_). The solution was 2.2 ppm Li as LiOH and 1200 ppm B as H_3_BO_3_ in weight. These test conditions such as temperature, pressure, and the content of Li and B were chosen to simulate a typical primary coolant in PWRs [24]. The exposure time for oxidation tests was selected to 500 h based on the experimental conditions of the previous studies regarding the corrosion behavior of nickel-based alloys such as Alloy 600 [16], Alloy 690 [6], and Alloy 182 [25].

The simulated primary water was stored in the solution tank with a capacity of 150 L. The DH contents were selected to 0.4494, 0.8988, 3.1458, 4.4940, 5.8422, and 8.9880 mg/kg (5, 10, 35, 50, 65, and 100 cc/kg H_2_O) at standard temperature and pressure (STP), which is a temperature of 0°C and a pressure of 1 bar by controlling the hydrogen overpressure of the solution tank. DO content was controlled in the range of 2–3 ppb to eliminate the oxygen effect on the corrosion potential and oxidation reaction. After the oxidation tests were finished, the specimens were directly cleaned in distilled water and dried in a vacuum oven at 60°C. Then, before analyzing the oxide layer by various analyses, the specimens were quickly stored in a vacuum desiccator at room temperature to avoid the variation on the oxide layer structure. In a previous study, the oxidation tests were conducted to Alloy 690TT under the same experimental conditions [22]. In this study, the oxidation tests were also carried out under the same conditions to clarify the relationship between oxide structure of Alloy 690TT and boron accumulation and the detailed chemical states of oxide layer by using XPS analysis, which was not performed in a previous study.

### 2.3. Analysis of Oxide Layer

The outer surface of the oxide layer formed on Alloy 690TT specimens was observed by using a SEM with Hitachi S-4800, of which acceleration voltage was 10 kV. The cross-section of oxide layer was also examined by using focused ion beam FIB (FEI company QUANTA 3D FEG)-SEM. The thickness of oxide layers was measured from the cross-sectional images obtained by FIB-SEM analysis. The thickness measurement was done at least at three different positions.

To elucidate the effect of oxide layer structure on the boron accumulation within the oxide, the depth profiles and chemical species of the oxide layers were analyzed by using XPS. The XPS analysis was carried out by using a Thermo Fisher Scientific (Theta Probe AR-XPS) X-ray photoelectron spectrometer with an Al K*α* X-ray source (1486.6 eV) operated at 15 kV and 150 W under a base pressure of 2.7 × 10^−7^ Pa. If necessary, the surface of the specimens was slightly etched using 1.0 kV argon ion beam. The binding energies of all peaks were corrected with the reference C1s peak at 284.5 eV.

The removal (ex situ) of Alloy 690TT specimen from the PWR primary water environment may result in a modification of the oxide layer structure. However, in previous studies [26, 27], there is no significant differences between in situ and ex situ characterization of surface oxide films of nickel-based alloys. Wang performed the in situ surface-enhanced Raman spectroscopy (SERS) to characterize oxide films formed on Alloy 600 and Alloy 690 in PWR primary water and compare the differences between in situ and ex situ characterizations of surface oxide films [26]. He presented that there is no significant changes to the oxide layer of Alloy 690 and only slight changes to the oxide layer of Alloy 600 are possible: partial conversion of Cr_2_O_3_ to *α*-CrOOH of Cr(OH)_3_ on cooling to room temperature, and a small increase in the relative amount of spinel upon exposure to air at room temperature [26]. Recently, Kim et al. [27] performed the in situ and ex situ Raman spectroscopy to analyze the oxide films formed on dissimilar metal weld (DMW) interfaces of nickel-based alloy/low alloy steel under hydrogenated high-temperature water condition. There was also no significant change in the oxide structure and only a slight difference in the intensity of the Fe_3_O_4_ and NiCr_2_O_4_ peaks [27].

## 3. Results and Discussion

### 3.1. SEM Analysis

**Figure 1:**
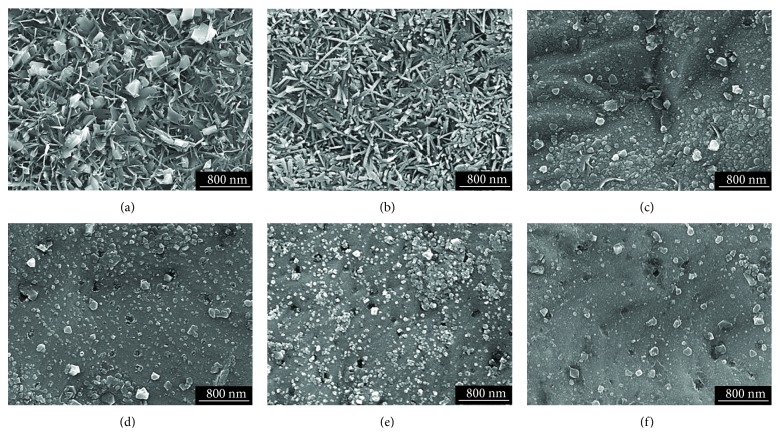
SEM micrographs of the oxide layers formed on Alloy 690TT in simulated primary water with different DH contents at 330°C for 500 h: (a) 0.4494 mg/kg, (b) 0.8988 mg/kg, (c) 3.1458 mg/kg, (d) 4.4940 mg/kg, (e) 5.8422 mg/kg, and (f) 8.9880 mg/kg.

Figure 1 shows the SEM micrographs of the oxide layers formed on Alloy 690TT in simulated PWR primary water with the broad DH range of 0.4494–8.9880 mg/kg. The morphologies of the external oxide layers were greatly changed corresponding to DH content. It was clear that the external oxide layer formed at DH = 0.4494 mg/kg was covered with numerous needle-like oxides and small planer oxide particles (Figure 1(a)). The needle-like oxide particles with a diameter of less than 100 nm were randomly tangled. The planer oxide particles were randomly dispersed with the needle-like oxides. The size of the planer oxide particles was approximately 150–400 nm. Whereas, a great number of needle-like oxides were only observed without the planar oxide particles on the surface of the specimen under the condition of DH = 0.8988 mg/kg (Figure 1(b)). The needle-like oxides at DH = 0.8988 mg/kg were much larger and thicker than the needle-like oxides at DH = 0.4494 mg/kg. As shown in Figures 1(c)–1(f), the morphology and number of the outer oxide particles formed at medium and high DH contents (DH = 3.1458–8.9880 mg/kg) are greatly different from the oxide particles formed at low DH contents (DH = 0.4494–0.8988 mg/kg). In the DH range of 3.1458–8.9880 mg/kg, the fine polyhedral oxide particles with a size of 20–200 nm were formed on the whole surface of specimens. The number of the oxide particles decreased with increasing the DH content.

**Figure 2:**
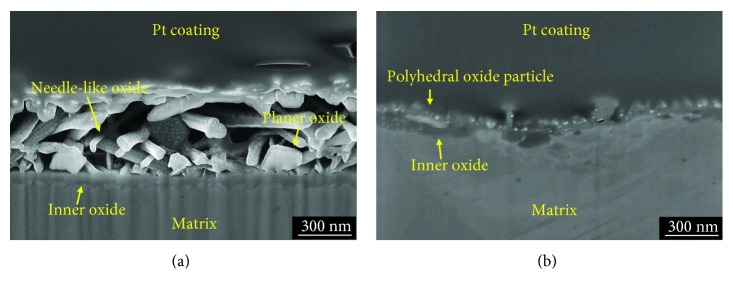
FIB-SEM micrographs of the oxide layers formed on Alloy 690TT in simulated primary water with different DH contents at 330°C for 500 h: (a) 0.4494 mg/kg and (b) 3.1458 mg/kg.

Figure 2 presents the FIB-SEM images of the cross-section of oxide layers formed under the conditions of DH = 0.4494 and 3.1458 mg/kg. In case of DH = 0.4494 mg/kg, the outer oxide layer had a thick and porous structure, whereas the inner oxide layer was compact and continuous. However, under the condition of DH = 3.1458 mg/kg, the external oxide layer had a thin and dense structure. In addition, the inner oxide layer appeared not to be continuous. The cross-sectional structure of the external oxide layers was well consistent with the top view images shown in Figure 2. The thickness of oxide layers decreased with increasing the DH content. The mean thickness of the oxide layer at DH = 0.4494 mg/kg was about 880 nm and approximately 185 nm at DH = 3.1458 mg/kg, respectively. In particular, the thickness of outer needle-like oxide at DH = 0.4494 mg/kg is very thick (about 800 nm).

### 3.2. XPS Depth Profile and Spectrum Analyses

**Figure 3:**
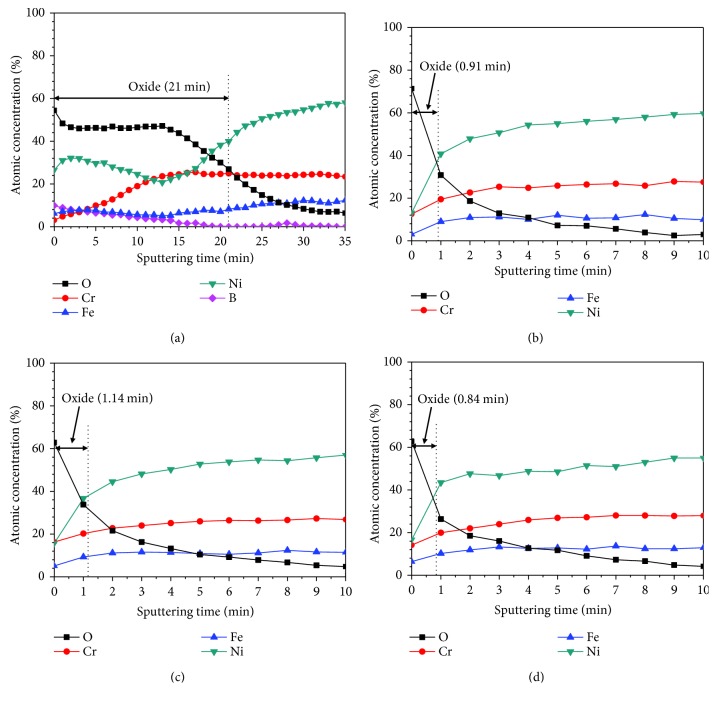
Depth profiles of the oxide layers formed on Alloy 690TT in simulated primary water with different DH contents at 330°C for 500 h: (a) 0.4494 mg/kg, (b) 3.1458 mg/kg, (c) 4.4940 mg/kg, and (d) 5.8422 mg/kg.

Figure 3 shows the XPS depth profiles of the oxide layers. From the half-height of the oxygen theory [14, 28], the thickness of oxide layers could be quantitatively estimated. That is, the point where the intensity of oxygen content reaches 50% of its initial value is taken as the interface between the oxide layer and steel substrate. The oxide layers formed at DH = 0.4494 mg/kg require approximately 21 min of sputtering time to reach 50% of its initial value. Under the conditions of DH = 3.1458, 4.4940 and 5.8422 mg/kg, the sputtering time of oxide films which the oxygen content was half of the initial value was about 1 min. Among the DH contents, the sputtering time of oxide layers at DH = 5.8422 mg/kg was the shortest about 0.84 min. These results indicate that the oxide layer formed at low DH content grew thicker, and the oxide layer formed at medium and high DH contents became thinner. These results could be supported by the fact the thickness of oxide layers decreased with increasing the DH content, which was observed in Figure 2. In addition, the B element content was detected about 10 at.% in the outermost NiO layer at DH = 0.4494 mg/kg (Figure 3(a)). In this layer, the content of B element in the NiO layer gradually decreased with the increase of sputtering time. Finally, the B element disappeared in the inner Cr-rich oxide layer. However, the B element was not detected under other DH contents (DH = 3.1458, 4.4940, and 5.8422 mg/kg).

In this work, the thickness of oxide layers could be associated with the thermodynamics and kinetics of corrosion and incorporation of boric oxide. In the viewpoint of corrosion thermodynamics, Cr_2_O_3_ and Fe_3_O_4_ are stable phases under all DH contents handled in this work. However, Ni/NiO phase transition is expected to be characterized by the effect of DH content on the electrochemical potential (ECP). The ECP of Alloy 690TT with various DH contents was calculated using Nernst equation for hydrogen-hydrogen ion exchange reaction, and the ECP gradually decreased with increasing DH content [22]. Alloy 690TT is located in the NiO stable region at low DH contents (DH = 0.4494–0.8988 mg/kg), whereas the stable state of Alloy 690TT is metallic Ni at medium and high DH contents (DH = 3.1458–8.9880 mg/kg) [22]. In the NiO stable region, Ni dissolution spontaneously occurs through an oxidative dissolution reaction as shown in the following reaction:

(1) $$\begin{array}{c} Ni\left(s\right)+H_{2}O↔NiO\left(s\right)+2H^{+}+2e^{-}. \end{array}$$

However, the oxidative dissolution reaction of Ni is reduced in the Ni stable region because Ni is no longer a thermodynamically oxidized state. Second, the corrosion kinetics is not only dependent on the microstructure and chemical composition but also dependent on the electronic property of oxide layer. These parameters are crucial to the stability of the oxide layers. Peng et al. [16] presented that increasing DH content decreases the ionic defect transport resistance in the oxide layer and leads to a more defective microstructure of the oxide layers using the in situ measurement of contact electric resistance (CER) and electrochemical impedance. They suggested that the increasing DH content leads to a decrease of the oxide layer thickness due to the differences of these corrosion kinetic parameters [16]. Third, the thickness of oxide layers formed on Alloy 690TT may be affected by the incorporation with boric oxide. Machet et al. [29] presented that a considerable amount of boric trioxide (B_2_O_3_) is incorporated in the oxide films formed on nickel-based alloy in simulated PWR primary water at 320°C and 155 bars. In this work, boric oxide could be incorporated in the oxide layer and results in a thicker oxide layer.

**Figure 4:**
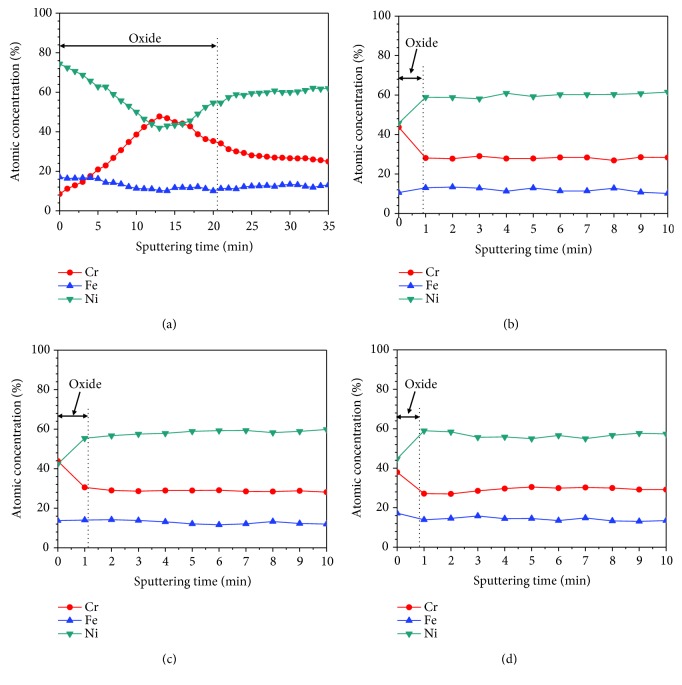
Normalization of the oxide films formed on Alloy 690TT in simulated primary water with different DH contents at 330°C for 500 h: (a) 0.4494 mg/kg, (b) 3.1458 mg/kg, (c) 4.4940 mg/kg, and (d) 5.8422 mg/kg.

The XPS depth profile of the three major elements (Ni, Cr, and Fe) normalized to 100% is also shown in Figure 4. As shown in Figure 4(a), at DH = 0.4494 mg/kg, the outer oxide layer is Ni-enriched while depletion of Cr is observed. With the increase of sputtering time, Ni dominant oxide layer disappeared, and the Cr content gradually increased to 48 at.%. Ni enrichment in the inner oxide layer occurred once again, and Cr content slightly decreased. The variation of Ni and Cr may be attributed to the effect of the chemical composition of the matrix. These depth profiles suggest that the outer oxide layer mainly consists of NiO layer, and the inner layer of the oxide layer is dominantly Cr_2_O_3_ layer. However, the distribution of elements exhibited a significant change in a range of DH = 3.1458–5.8422 mg/kg. The distribution of all elements is very similar under these DH conditions. As shown in Figures 4(b)–4(d), Cr is enriched while Ni is depleted in the outer oxide layer. The increase of Ni content and decrease of Cr content were observed from the surface to the oxide/substrate interface with increasing sputtering time. The variation of Ni and Cr may be due to the matrix.

**Figure 5:**
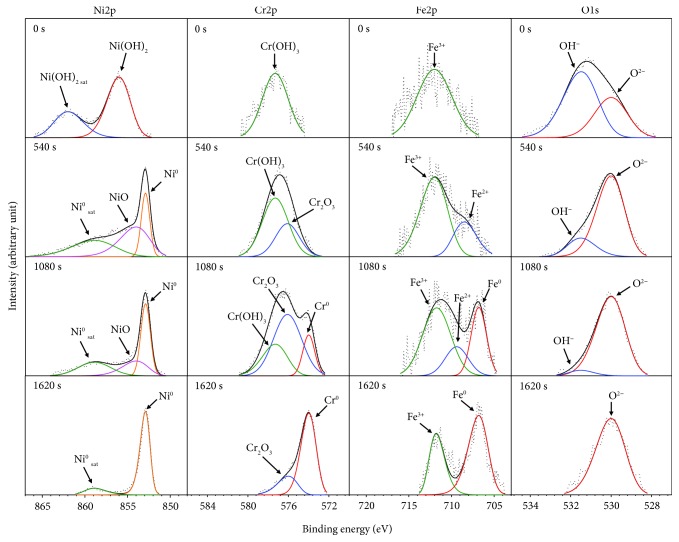
XPS spectra of Ni, Cr, Fe, and O for the oxide layers formed on Alloy 690TT in simulated primary water with DH = 0.4494 mg/kg at 330°C for 500 h.

Figure 5 shows the XPS spectra of Ni 2p_3/2_, Cr 2p_3/2_, Fe 2p_3/2_, and O 1s for the oxide layers formed at DH = 0.4494 mg/kg. The binding energies (*E*_b_) obtained from the deconvoluted XPS profiles of the primary compounds in the layers are listed in Table 2. First, the XPS spectra of Ni 2p_3/2_ show that a peak of Ni(OH)_2_ and a satellite peak of Ni(OH)_2_ could be observed at the outermost oxide surface. At the sputtering time of 540 s, a Ni^0^ peak and a satellite Ni^0^ peak were observed in addition to the NiO peak, while the Ni(OH)_2_ peaks disappeared. Following a sputtering time of 1080 s, an increase of Ni^0^ and decrease of NiO peak intensities were observed. At the final sputtering time of 1620 s, only the Ni^0^ peak was identified. The formation of Ni(OH)_2_ at the outer oxide layer is most likely owed to the hydration of Ni^2+^. Second, the Cr 2p_3/2_ spectrum exhibits a peak related to Cr(OH)_3_ on the outermost surface of the oxide layer. Underneath the surface layer, the Cr 2p_3/2_ spectrum exhibits a Cr_2_O_3_ and Cr(OH)_3_ peak at the sputtering time of 540 s. At the sputtering time of 1080 s, an increase of the Cr_2_O_3_ peak intensity and a decrease of the Cr(OH)_3_ peak intensity were observed. The peak corresponding to Cr^0^ was also observed. Further increasing the sputtering time to 1620 s, a significant increase of the Cr^0^ peak intensity and a decrease of Cr_2_O_3_ peak intensity were observed in association with the disappearance of the Cr(OH)_3_ peak. Third, only the Fe^3+^ peak was observed at the outermost surface of the oxide layer. However, following a 540 s sputtering, the Fe^2+^ peak was observed in addition to the Fe^3+^ peak. Increasing the sputtering time to 1080 s resulted in the detection of Fe^0^, along with Fe^2+^ and Fe^3+^ peaks. At the final sputtering time of 1620 s, Fe^2+^ peak was not observed, while the increase of Fe^0^ peak and decrease of Fe^3+^ peak could be identified. Finally, the XPS spectra of O 1s show that two peaks could be identified at the outermost surface of the oxide layer (O^2−^ and OH^−^ peaks). Increasing the sputtering time to 540 s and further to 1080 s led to enhanced O^2−^ but weakened OH^−^ peaks. Only O^2−^ peak was observed at the final sputtering times of 1620 s. This was consistent with the above results that the inner layer of the oxide layer consisted of Cr_2_O_3_ peak, while the surface of the external oxide layer was segregated by Ni(OH)_2_ and Cr(OH)_3_ due to the hydration of Ni^2+^ and Cr^3+^.

**Figure 6:**
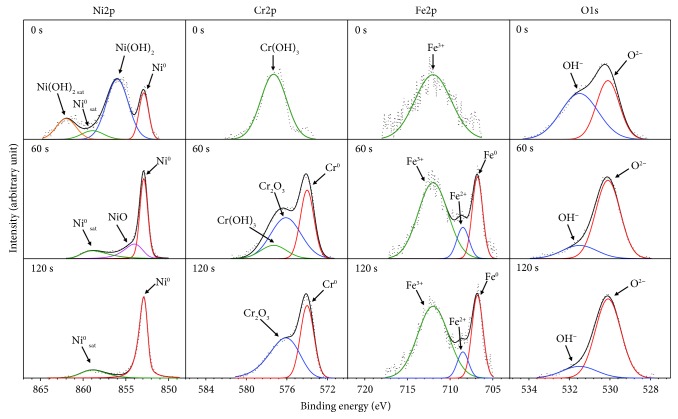
XPS spectra of Ni, Cr, Fe, and O for the oxide layers formed on Alloy 690TT in simulated primary water with DH = 4.4940 mg/kg at 330°C for 500 h.

Figure 6 shows the XPS spectra of Ni 2p_3/2_, Cr 2p_3/2_, Fe 2p_3/2_, and O 1s for the oxide layers formed at DH = 4.4940 mg/kg. First, Ni 2p_3/2_ spectrum exhibits the peak of Ni^0^ and peak of Ni(OH)_2_ at the outer oxide surface. At the sputtering time of 60 s, the increased Ni^0^ peak was observed in addition to the NiO peak, while the Ni(OH)_2_ peak disappeared. At the final sputtering time of 120 s, the Ni^0^ peak was only observed. Second, the XPS spectra of Cr 2p_3/2_ show only a Cr(OH)_3_ peak could be examined at the outer surface of the oxide layer. At the sputtering time of 60 s, the weakened Cr(OH)_3_ peak could be detected in addition to the Cr^0^ peak and Cr_2_O_3_ peak. The increase of the Cr^0^ and Cr_2_O_3_ peak intensity in association with the disappearance of the Cr(OH)_3_ peak was observed at the sputtering time of 120 s. Third, Fe 2p_3/2_ spectrum shows the Fe^3+^ peak was only observed on the outermost surface of the oxide layer. At the sputtering time of 60 and 120 s, the Fe^3+^ peak was observed along with Fe^0^ peak and Fe^3+^ peak. Finally, the XPS spectra of O 1s show that O^2−^ peak and OH^−^ peak could be observed at the surface of the oxide layer. Increasing the sputtering time to 60 s and further to 120 s led to enhanced O^2−^ but weakened OH^−^ peaks.

**Figure 7:**
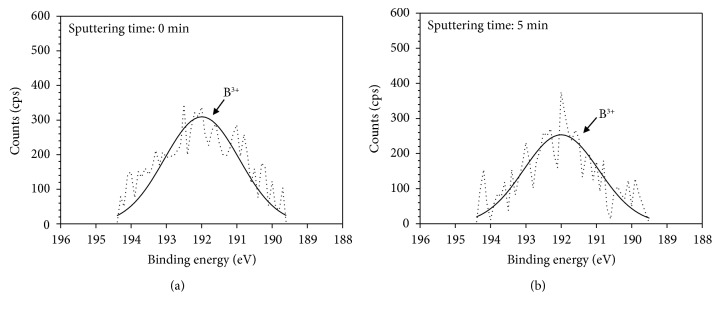
XPS spectra of B1s for the oxide layers formed on Alloy 690TT in simulated primary water with DH = 0.4494 mg/kg at 330°C for 500 h (a) without sputtering and (b) with sputtering time for 300 s.

Figure 7 presents the XPS spectra of B 1s for the oxide layers formed at DH = 0.4494 mg/kg. As shown in Figure 7(a), B 1s spectrum exhibits the peak of B^3+^ at the outer oxide surface. At the sputtering time of 300 s, the slightly weakened peak of B^3+^ was still observed within the oxide layer (Figure 7(b)). However, the peak of B^3+^ was not detected at DH = 4.4940 mg/kg. These results indicate that the boron compounds containing B^3+^ were accumulated within the thick and porous NiO layer at low DH contents, whereas the accumulation of boron compounds did not occur in the dense and thin polyhedral (Ni, Cr, and Fe)O layer at medium and high DH contents.

**Figure 8:**
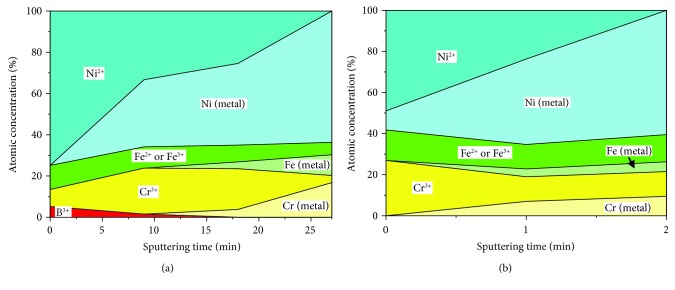
Speciated chemical composition profiles of the oxide layers formed on Alloy 690TT with sputtering time in simulated primary water with various DH contents at 330°C for 500 h: (a) 0.4494 mg/kg and (b) 4.4940 mg/kg.

Based on the deconvoluted XPS profiles, the depth profile of chemical species on the oxide layers could be presented.  Figure 8 shows the depth profile of chemical species on the oxide layers formed at DH = 0.4494 and 4.4940 mg/kg. As mentioned above, the outer part of the oxide layer formed at DH = 0.4494 mg/kg consisted predominantly of Ni^2+^ (Figure 8(a)). By contrast, Cr^3+^ and Ni^0^ were primarily located in the inner part of the oxide layer under this condition. These results indicate that the double-structured oxide layers composed of the outer NiO layer and inner Cr_2_O_3_ layer were formed at DH = 0.4494 mg/kg. In the oxide layer formed at DH = 4.4940 mg/kg, the outer part of the oxide layer predominantly consisted of Ni^2+^ and Cr^3+^. The Ni^2+^ and Cr^3+^ content gradually decreased, and Ni^0^ content increased with the increase of sputtering time (Figure 8(b)). Finally, the Ni^0^ dominated in the inner part of the oxide layer. These results can indicate that the oxide layer formed at DH = 4.4940 mg/kg consists of outer spinel oxides such as NiCrFeO_4_ and metallic Ni-rich layer. However, in this case, the inner Cr_2_O_3_ layer was not detected because the inner Cr_2_O_3_ layer appeared not to be continuous.

According to the XPS results, the oxide layers formed on Alloy 690TT were composed of oxides and hydroxides under all hydrogen conditions. The formation of Ni(OH)_2_ and Cr(OH)_3_ at the outer oxide layer is most likely due to the hydration of Ni^2+^ and Cr^3+^. The oxide layers at low DH content were composed of an outermost layer of Ni(OH)_2_ and Cr(OH)_3_ enriched in Ni, an intermediate layer of hydroxides and oxides enriched in Cr, and an inner Cr_2_O_3_ layer. Outermost NiO coexists with a small amount of Cr_2_O_3_ layer; while in the inner oxide, only Cr_2_O_3_ remains. The oxide layers at medium and high DH contents were composed of an outermost layer of Ni(OH)_2_ and Cr(OH)_3_ enriched in Cr, an intermediate layer of metallic Ni, hydroxides and oxides enriched in Cr, and an inner Cr_2_O_3_ layer.

### 3.3. Effect of Oxide Layer Structure on Boron Accumulation

Boron, in the form of boric acid (H_3_BO_3_), is added into primary water to control the neutron flux while lithium hydroxide (LiOH) is also dosed to control the pH of the primary water. As presented in the EPRI report [38], boron can accumulate in the pores of crud on the fuel cladding as a concentrated solution or a solid phase when the crud was built upon cladding.

Previous studies have reported that there are only a few different forms of boron species in fuel crud originating from PWR core. It is well known that boric acid may be thought of as hydrates of boric trioxide (B_2_O_3_). Doncel et al. [39] presented that boron-containing compound is crystalline lithium tetraborate (Li_2_B_4_O_7_), which previously has been identified in simulated fuel crud. Recently, nickel-iron oxyborate known as bonaccordite- (Ni_2_FeBO_5_-) containing boron was discovered and characterized in fuel crud samples taken from some high fuel-duty PWRs [40]. However, it is not yet known in what form boric acid or oxide is deposited in a PWR fuel crud, although some researchers believe that a predominant form of boron in axial offset anomaly (AOA) fuel crud is lithium metaborate (LiBO_2_) [41].

**Figure 9:**
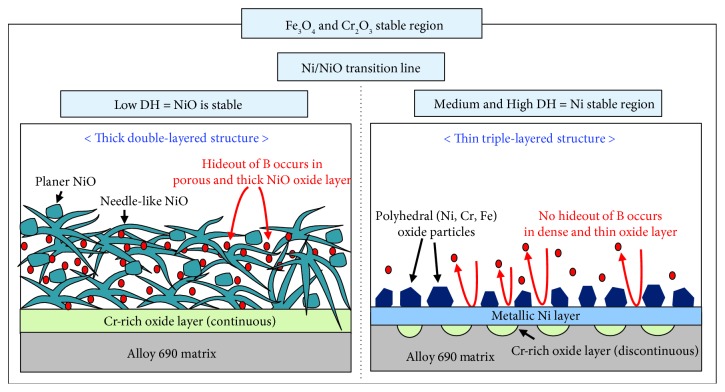
Schematic of the effects of DH contents on the oxide layer structure and associated boron accumulation of Alloy 690TT in simulated primary water at 330°C.

In this study, to elucidate the relationship between the oxide layer structure and boron accumulation within the oxide layers, the depth profiles and chemical species of the oxide layers formed on Alloy 690TT were analyzed by using XPS. It was observed that the B^3+^ ions were detected in the thick and porous NiO layer formed at low DH contents. This indicates that the boron compounds containing B^3+^ ions were accumulated in the thick and porous NiO layer. The content of B^3+^ ions gradually decreased with the increase of sputtering time. Finally, the B^3+^ ions disappeared in the inner Cr_2_O_3_ layer. However, the accumulation of B^3+^ ions did not occur in the dense and thin polyhedral NiCrFeO_4_ layer formed at medium and high DH contents (DH = 3.1458–8.9880 mg/kg). Based on the results, it is confirmed that the morphology and thickness of oxide layer formed on Alloy 690TT could significantly affect the accumulation of boron compounds within its oxide layer. The effect of DH content on the oxide layer structure of Alloy 690TT and associated boron accumulation within its oxide layer is schematically shown in Figure 9.

According to EPRI report, the DH content has been specified to be controlled in a range of 2.2470–4.4940 mg/kg in the primary water chemistry guidelines [24]. EPRI report showed that the DH contents (0.4494– 5.2130 mg/kg) do not appear to greatly impact the relative boron uptake within the crud of fuel cladding in simulated primary water of PWRs, although some systematic trends in the chemical composition of the crud resulting from change in DH contents could be detected [34]. In addition, several researchers suggest that the content of hydrogen does not greatly affect the amount of crud deposits, boron accumulation, and corrosion resistance of fuel cladding [18, 19]. However, Baek et al. [19] proposed that nuclear power plants should be operated at high DH content to prevent the formation of whisker-structured crud on the fuel cladding and thus mitigate the AOA problem.

We think that boron accumulation could occur within not only the pores of crud on the fuel cladding but also porous oxide layer formed on the Alloy 690TT at low DH contents (0.4494–0.8988 mg/kg) because both oxide layers are porous and thick. Based on the results, it was clear that boron compounds containing B^3+^ were accumulated in the thick and porous NiO layer formed at low DH contents.

Under the operation of nuclear power plants, the accumulation of boron compounds containing B^3+^ ions within the oxide layer of Alloy 690TT should be considered for establishing the operation condition of hydrogen content. Accumulation of boron compounds could accelerate general corrosion and primary water stress corrosion cracking (PWSCC) due to a decrease in pH within the porous oxide layer as through the following precipitation reaction of LiBO_2_.

(2) $$\begin{array}{c} {Li}^{+}+H_{3}{BO}_{3}↔{LiBO}_{2}+H^{+}+H_{2}O. \end{array}$$

Based on the results obtained from this work, it is concluded that the medium and high DH contents located in the Ni stable region are more desirable for SG tube for prohibiting the boron accumulation within the oxide layer of Alloy 690TT because the hideout of boron compounds does not occur in the external dense and thin oxide layer.

## 4. Conclusions

1. In primary water of PWRs at 330°C, the oxide layers at low DH content were composed of an outermost layer of Ni(OH)_2_ and Cr(OH)_3_ enriched in Ni, an intermediate layer of hydroxides and oxides enriched in Cr, and an inner Cr_2_O_3_ layer. Outermost NiO coexists with a small amount of Cr_2_O_3_ layer, while in the inner oxide only Cr_2_O_3_ remains.
2. The oxide layers at medium and high DH content were composed of an outermost layer of Ni(OH)_2_ and Cr(OH)_3_ enriched in Cr, an intermediate layer of metallic Ni, hydroxides and oxides enriched in Cr, and an inner Cr_2_O_3_ layer.
3. By XPS analysis, it was observed that B^3+^ ions were accumulated in the porous NiO layer formed at low DH content, whereas the accumulation of B^3+^ ions did not occur in the dense polyhedral oxide layer at medium and high DH contents.
4. Boron compound accumulation should be considered within not only crud on fuel cladding but also oxide layer on SG tube owing to corrosion problem and the content of boron in primary coolant of PWRs.

## Figures and Tables

**Table 1:** Chemical composition of Alloy 690TT (wt. %).

C	Cr	Fe	Si	Mn	Ti	Al	Ni
0.02	28.0	10.2	0.1	0.3	0.1	0.1	Bal.

**Table 2:** The binding energies of some chemical species for the XPS analysis.

Chemical species	Binding energies (eV)	Chemical species	Binding energies (eV)
Ni^o^_2p3/2_	852.9 [30–33]	Fe^o^_2p3/2_	706.8 [34–36]
Ni^o^_sat 2p3/2_	858.9 [30–33]	Fe^2+^_2p3/2_	708.5 [34–36]
NiO _2p3/2_	854.0 [30–33]	Fe^3+^_2p3/2_	712.0 [34–36]
Ni(OH)_2 2p3/2_	856.0 [30–33]		
Ni(OH)_2 sat 2p3/2_	862.0 [30–33]	O^2−^_1s_	530.0 [30–33]
		OH^−^_1s_	531.5 [30–33]
Cr^o^_2p3/2_	574.0 [30–33]		
Cr_2_O_3 2p3/2_	576.1 [30–33]	B^3+^_1s_	192.0 [37]
Cr(OH)_3 2p3/2_	577.3 [30–33]		
